# Developmentally Driven Changes in Adipogenesis in Different Fat Depots Are Related to Obesity

**DOI:** 10.3389/fendo.2020.00138

**Published:** 2020-03-26

**Authors:** Jana Breitfeld, Stephanie Kehr, Luise Müller, Peter F. Stadler, Yvonne Böttcher, Matthias Blüher, Michael Stumvoll, Peter Kovacs

**Affiliations:** ^1^University of Leipzig Medical Center, IFB Adiposity Diseases, University of Leipzig, Leipzig, Germany; ^2^Bioinformatics Group, Department of Computer Science and Interdisciplinary Center for Bioinformatics, University of Leipzig, Leipzig, Germany; ^3^Medical Department III—Endocrinology, Nephrology, Rheumatology, University of Leipzig Medical Center, Leipzig, Germany; ^4^Max Planck Institute for Mathematics in the Sciences, Leipzig, Germany; ^5^German Centre for Integrative Biodiversity Research (iDiv) Halle-Jena-Leipzig, Leipzig, Germany; ^6^Facultad de Ciencias, Universidad National de Colombia, Bogotá, Colombia; ^7^Santa Fe Institute, Santa Fe, NM, United States; ^8^Institute for Theoretical Chemistry, University of Vienna, Vienna, Austria; ^9^Institute of Clinical Medicine, University of Oslo, Oslo, Norway; ^10^Department of Clinical Molecular Biology, Akershus Universitetssykehus, Lørenskog, Norway

**Keywords:** adipogenesis, transcriptome, adipocyte, mRNA, gene expression, TTCA

## Abstract

Subcutaneous (sc) and visceral (vis) adipose tissue (AT) contribute to the variability in pathophysiological consequences of obesity and adverse fat distribution. To gain insights into the molecular mechanisms distinguishing vis and sc fat, we compared the transcriptome during differentiation of immortalized adipocytes from murine epididymal (epi) and inguinal (ing) AT. RNA was extracted on different days of adipogenesis (−2, 0, 2, 4, 6, 8) and analyzed using Clariom™ D mouse assays (Affymetrix) covering >214,900 transcripts in >66,100 genes. Transcript Time Course Analysis revealed 137 differentially expressed genes. The top genes with most divergent expression dynamics included developmental genes like *Alx1, Lhx8, Irx1/2, Hoxc10, Hoxa5/10*, and *Tbx5/15*. According to pathway analysis the majority of the genes were enriched in pathways related to AT development. Finally, in paired samples of human vis and sc AT (*N* = 63), several of these genes exhibited depot-specific variability in expression which correlated closely with body mass index and/or waist-to-hip ratio. In conclusion, intrinsically programmed differences in gene expression patterns during adipogenesis suggest that fat depot specific regulation of adipogenesis contributes to individual risk of obesity.

## Introduction

Obesity increases the individual risk for type 2 diabetes (T2D), dyslipidaemia, fatty liver disease, hypertension, and cardiovascular disease (CVD) (1, 2). However, obesity itself does not necessarily lead to these comorbidities (3), although adipose tissue (AT) distribution may contribute to cardio-metabolic diseases even in lean individuals (4). It is well-established that fat stored in visceral (vis) depots makes individuals more prone to metabolic complications than subcutaneous (sc) fat (5–7).

Given the association with various metabolic disorders, understanding the underlying molecular mechanisms of AT heterogeneity and body fat distribution (FD) has emerged to be one of the greatest challenges in obesity research (8). It has been shown that genes differentially expressed in various AT depots represent plausible candidates involved in the regulation of body FD (9–11). In particular, there is clear evidence for depot specific expression of developmental genes like *Short Stature Homeobox 2* (*Shox2*), *T-Box 15* (*Tbx15*), *Engrailed 1* (*En1*), and Homeobox-genes (e.g., *Hoxa5, Hoxc8*, and *Hoxc9*) which have been suggested to play a role in the origin of obesity and body FD (9). Moreover, recent genome-wide association studies (GWAS) suggested genes involved in adipogenesis to be attractive candidates potentially regulating body FD (12).

Although under general debate at the cellular level, vis AT contains larger cells and lower numbers of adipocytes compared to sc AT (8, 13). A higher insulin-sensitivity and avidity for free fatty acid (FFA) and triglyceride (TG) uptake is characteristic for smaller adipocytes (14, 15) which could partially explain the beneficial effects of sc AT (16). Moreover, vis adipocytes manifest a higher catecholamine-induced lipolytic activity (17, 18) and are less sensitive to the anti-lipolytic action of insulin. From the molecular point of view, expression of receptors and secretion of adipokines belong to the major players highlighting differences of AT depots (19). Activation of adipocyte receptors can occur by circulating endocrine hormones, by adipokines secreted from neighboring adipocytes exerting paracrine effects and by signals mediated via the central nervous system (18). Adipokines, which are highly active proteins secreted by adipocytes, have been extensively studied during the last decades due to their prominent anti- and pro-inflammatory effects associated with obesity (20–22). In addition, increased vis AT mass and insulin resistance (23) appear to be crucial players linking abdominal vis adiposity to CVD (24). Attributed to their close relationship with clinical phenotypes, the physiological and metabolic features of the respective regional fat depots may serve as tools in the clinical practice for predicting increased risks for the development of T2D, metabolic syndrome and CVD (25–27).

Investigating differences between vis and other AT depots such as sc AT is therefore inevitable not only to elucidate genes controlling body FD but also to better understand the specific mechanisms by which vis fat exerts its detrimental metabolic effects or why sc fat may appear beneficial. To uncover novel genes potentially regulating FD and to shed light on potentially distinct depot-specific pathways contributing to the known differences distinguishing vis and sc fat, in the present study, we employed a comprehensive genome-wide mRNA expression profiling to identify differentially regulated genes in the course of adipogenesis in murine epididymal (epi; corresponding to human vis AT) vs. inguinal (ing; corresponding to human sc AT) immortalized adipocytes (28, 29). Moreover, we tested in human AT whether the identified genes exhibit fat-depot related changes in expression that correlate with the degree of obesity and the pattern of FD.

## Materials and Methods

### Cell Culture

Dipose tissue of newborn FVB mice was extracted and immortalized using the SV40 T antigen as described in detail elsewhere (28). These immortalized epi and ing adipocytes were cultured and differentiated according to the reported protocols (28, 29). Shortly, cells were grown in Dulbecco's modified Eagle's medium (high glucose) supplemented 20% fetal bovine serum at 37°C and 5% CO_2_ until reaching 80% confluence. Induction was then initiated by adding 0.125 mM indomethacine, 2 μg/ml dexamethasone, and 0.5 mM isobutylmethylxanthine to the growth medium for 24 h and for differentiation, growth medium was supplemented with 20 mM insulin and 1 nM triiodthyronine. Subsequently, cells were grown for 8 days in differentiation medium. Thus, cells were harvested at time point: 80% confluence (= day −2), day 0 (= day of induction), day 2, 4, 6, 8 (= 2, 4, 6, 8 days after induction), washed and frozen immediately at −80°C until RNA extraction. All differentiation lines were run in triplicates and for each time point three samples were collected. Adipocyte differentiation was monitored by AdipoRed™ staining [Supplemental Figure 1 (Supplement Tables and Figures are located at: http://www.bioinf.uni-leipzig.de/supplements/18-054)].

### RNA Extraction and Gene Chip Analysis

Total RNA was extracted using TRIzol (Life Technologies, Grand Island, NY, USA) according to the manufacturer's protocol. RNA samples at each time point were further pooled for each single differentiation line. The triplicates were analyzed using Clariom™ D mouse assays (Applied Biosystems™, Thermo Fischer Scientific) which cover >214,900 transcripts in >66,100 genes (*N* = 3 for each time point). Gene expression arrays were processed and analyzed in the Core Unit DNA–Technologies (Dr. Knut Krohn, University of Leipzig).

### Pre-Processing

The raw microarray data (cel-files) were corrected for background, log_2_-transformed and normalized using the standard method RMA (robust multi-array average) (Supplemental Table 1). Afterwards detection above background *p*-values were computed and genes were considered expressed, if half of its exons were expressed in at least one sample with a detection above background *p* < 0.05 (Supplemental Table 2). The pre-processing was done with the software package APT (affymetrix power tools) and the R Bioconductor package oligo (30–33).

### Transcript Time Course Analysis (TTCA)

We used the newly introduced Transcript Time Course Analysis (TTCA) (34), to identify genes that are differentially expressed between epi and ing AT over time. TTCA is considering not only comparisons at single time point (Supplemental Method 1 and Supplemental Table 3) but gene expression dynamics over the whole measured differentiation period. TTCA computes four scores that emphasize different aspects in gene expression dynamics during a time-course experiment: (i) the dynamic score reflects slow, constant changes in gene expression dynamics; (ii) the peak score emphasizes fast transient expression changes; and (iii) the integral score highlights absolute expression changes in different time intervals. Here various time intervals were considered: the early, the middle, the late, and the whole period of the experiment. We defined the early interval, as the time before the differentiation [80% confluence (day −2) to day of induction (day 0)], the middle interval as the 4 days following differentiation (day 0–day 4), and day 6–day 8 represented the late interval. Furthermore, (iv) a relevance score contains the log-transformed number of publications found in Pubmed that associate the gene name with a provided stimuli. In this analysis we defined the term “fat distribution” as stimulus. To ensure that the relevance score is not biasing the results toward detection of genes well studied in the context of fat distribution, we checked the correlation between consensus score and relevance score. The Pearson correlation coefficient is 0.23, and genes with high consensus scores include both, genes with many co-occurrences with the term “fat distribution” in Pubmed, as well as completely novel candidate genes in this context (Supplemental Figure 2). Additionally, TTCA checks for significant instability, so intersample variance within the gene expression intensities.

Last, TTCA combines all scores to a consensus score, by averaging all four scores and scaling the results between 0 and 1. The result-table obtained from TTCA contained 4,797 genes significant in at least one of the computed scores (Supplemental Table 4). For further analysis we focused on 137 genes significant in all four scores without showing significant instability (Supplemental Table 5).

### Gene Set Enrichment Analysis (GSEA)—GO-Term and KEGG Pathway Analysis

We performed a gene set enrichment analysis (GSEA) to identify enriched processes, functions, and pathways within the set of genes being differentially expressed during differentiation in epi and ing adipocytes. For analyses we used the web-based resources GOrilla [http://cbl-gorilla.cs.technion.ac.il/; (35)], DAVID [https://david.ncifcrf.gov/home.jsp (36)], and StringDB [https://string-db.org/ (37)].

Enriched KEGG pathways (Kyoto Encyclopedia of Genes and Genomes) (38) were extracted from the DAVID database. For advanced visualization, considering also the differential gene expression dynamics we computed log_2_ fold changes (log_2_-FC) between epi and ing expression values for each measured time-point. The enriched KEGG pathways were visualized using the R bioconductor package pathview (39). As prerequisite the Affymetrix probeset IDs have been mapped to ENTREZ-IDs by means of the R packages affycoretools, AnnotationDbi, org.Mm.eg.db, and mta10sttranscriptcluster.db (40–42).

### Categorized Congruent Gene Dynamics

In a first step we clustered all genes by k-means (43). In contrast to other approaches, we did not separate the different conditions (here epi and ing) or concatenate their profiles. As we were interested in the information of whether the expression of a gene is constant, decreasing or increasing, we used the vector of slopes (or derivation) for clustering. The slope of the gene expression intensity x at time point i is approximated by $x_{i}^{\prime }\approx \frac{x_{i+1}-x_{i-1}}{2^{*}\left(i+1\right)-\left(i-1\right)}$. The number of clusters was defined as *k* = 8. The choice is based on the observation of the ratio of between sum of squares / total sum of squares, rating the similarities of genes within one cluster and the overall similarity of genes (data not shown).

In a second step, for each gene, we extracted the occurring cluster combinations representing the gene expression pattern in epi vs. ing. If a gene was categorized to the same cluster in both tissues, the gene expression dynamics was similar, although the expression intensity varies. The gene responded to the differentiation signal with different expression dynamics if the according epi and ing profiles fell into different clusters.

### mRNA Expression in Human Adipose Tissue

To validate the identified top candidate genes in human AT, we used a previously generated dataset which allowed extracting mRNA expression levels for individually selected genes.

Briefly, paired samples of vis and sc AT were taken from patients who underwent open abdominal surgery for e.g., cholecystectomy or weight reduction surgery as described elsewhere (44). The present study included 63 subjects who have previously undergone mRNA expression profiling as reported in detail elsewhere (44). The participants were lean (*N* = 25), sc obese (*N* = 21), or vis obese (*N* = 17), which was defined by the ratio of vis to sc AT (45). Thus, a cut-off <0.4 defined sc and the cut-off >0.4 vis obese individuals (45). Further, the mean age was 53 ± 16 years, the mean body-mass index (BMI) 36.1 ± 14.0 kg/m^2^ and the mean waist-to-hip ratio (WHR) 0.953 ± 0.149. Correlation analysis of gene expression levels with obesity measures was performed using IBM SPSS Statistics 24 software (IBM Corp., Armonk, NY, USA). Since the phenotypic data were not normally distributed (even after ln transformation), Spearman's correlation was conducted to assess the relationship between mRNA expression and BMI as well as WHR. Linear regression analyses were adjusted for age, and gender. Comparisons between groups were done by Mann-Whitney-test and *P* ≤ 0.05 were considered to be significant.

## Results

### Differential Gene Expression Analysis During Adipogenesis

**Figure 1:**
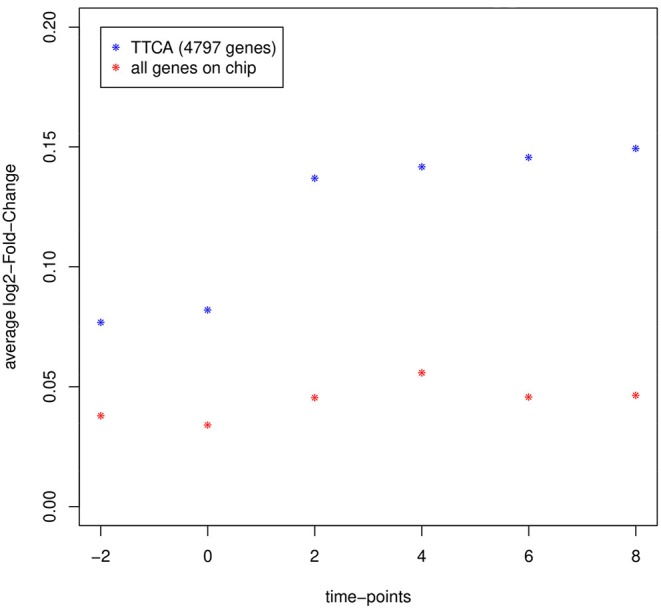
Average log_2_-fold-change of gene expression intensity between epididymal and inguinal at each measured time point of all genes on the chip considered for analysis (red; *N* = 62,349) compared to genes reported as differentially expressed by TTCA (blue; *N* = 4,797). Gene expression intensities in both types of adipocytes diverge after the differentiation stimulus at day 0 and TTCA is able to select genes with highly different expression in the experiment.

To evaluate gene expression differences between the two depots, as well as the effectiveness of the TTCA method, we explored the overall global change of gene expression at each time point. Subsequently, the average log_2_-FC of all genes on the chip was compared with the log_2_-FC of the 4,797 genes reported as differentially expressed by TTCA (Figure 1).

Indeed, the method was proved to be valid to detect genes responding to the differentiation stimulus at day 0. As expected the expression differences between the two fat cell-types increased, reflecting the growing molecular divergence between epi and ing (Figure 1).

Following the TTCA which provided a list of genes reported as significant in at least one of the four scores (Supplemental Table 4), we filtered those genes that were significant in all computed scores and did not show significant instability i.e., variability between replicates. The 137 remaining genes (Supplemental Table 5) showing the most prominent differences during adipogenesis between epi and ing adipocytes were taken forward for a GSEA (see details below). Based on the distribution of the consensus scores of the filtered genes, all genes within the upper quartile (consensus score > 0.5607811 cutoff; Supplemental Figure 3), were defined as the top 34 genes with most divergent expression dynamics during adipogenesis in epi vs. ing cell lines (Table 1, Supplemental Table 5).

**Table 1:** Thirty-four most differentially expressed genes between epididymal and inguinal adipocyte differentiation over 8 days.

**Gene symbol**	**Name**	**Consensus score**
*Alx1*	alx homeobox gene 1	1.000
*Lhx8*	LIM homeobox 8	0.878
*Ptn*	pleiotrophin	0.845
*Epha3*	EPH receptor A3	0.796
*Dkk2*	Dickkopf WNT signaling pathway inhibitor 2	0.793
*Gatm*	Glycine Amidinotransferase	0.755
*Negr1*	Neuronal growth regulator 1	0.754
*Gm24598 (SNORD113/SNORD114–RF00181)*	box C/D snoRNA SNORD113/SNORD114	0.733
*Cd36*	CD36 molecule (thrombospondin receptor)	0.730
*Apod*	Apolipoprotein D	0.714
*Tbx5*	T-Box 5	0.697
*Hoxc10*	Homeobox C10	0.685
*Hoxa5*	Homeobox A5	0.673
*NONMMUT007163, NONMMUT007164, NONMMUT007165*	Non-coding antisense	0.663
*NONMMUT015745, NONMMUT015746, NONMMUT015747*	lincRNA	0.657
*Irx1*	Iroquois homeobox protein 1	0.655
*Ace*	Angiotensin I converting enzyme	0.653
*Dlk1*	Delta like non-canonical notch ligand 1	0.651
*Adh7*	Alcohol dehydrogenase 7	0.650
*Irx2*	Iroquois homeobox protein 2	0.645
*9930111J21Rik2*	RIKEN cDNA 9930111J21 gene 2	0.640
*Arhgdib*	Rho GDP Dissociation Inhibitor Beta	0.639
*Gm4955*	Interferon-activated gene 206	0.639
*Mmp2*	Matrix Metallopeptidase 2	0.636
*Tgtp2*	T-cell-specific guanine nucleotide triphosphate-binding protein 2	0.625
*Hoxa10*	Homeobox A10	0.616
*9930111J21Rik1*	RIKEN cDNA 9930111J21 gene 1	0.610
*Cidec*	Cell death inducing DFFA like effector C	0.607
*KnowTID_00007994*	lincRNA	0.606
*Eif2s3y*	Eukaryotic translation initiation factor 2. subunit 3	0.604
*Oas2*	2'-5'-Oligoadenylate Synthetase 2	0.594
*Tnfrsf26*	Tumor necrosis factor receptor superfamily, member 26	0.592
*Gbp6*	Guanylate-binding protein 10	0.582
*Tbx15*	T-Box 15	0.563

### Cluster Analysis

In the initial k-means clustering step the gene expression slopes (see methods for details) of the 137 most prominent differentiating genes were assigned to eight different clusters. Each of the eight clusters can be translated into a schematic gene expression pattern considering the following rules:

1. positive slope at time-point i (x${}_{\text{i}}^{\prime }$ > 0) → gene expression increases (rise);
2. slope at time-point i is zero (x${}_{\text{i}}^{\prime }$ = 0) → constant gene expression or change of direction (constant);
3. negative slope at time-point i (x${}_{\text{i}}^{\prime }$ < 0) → gene expression decrease (fall).

**Figure 2:**
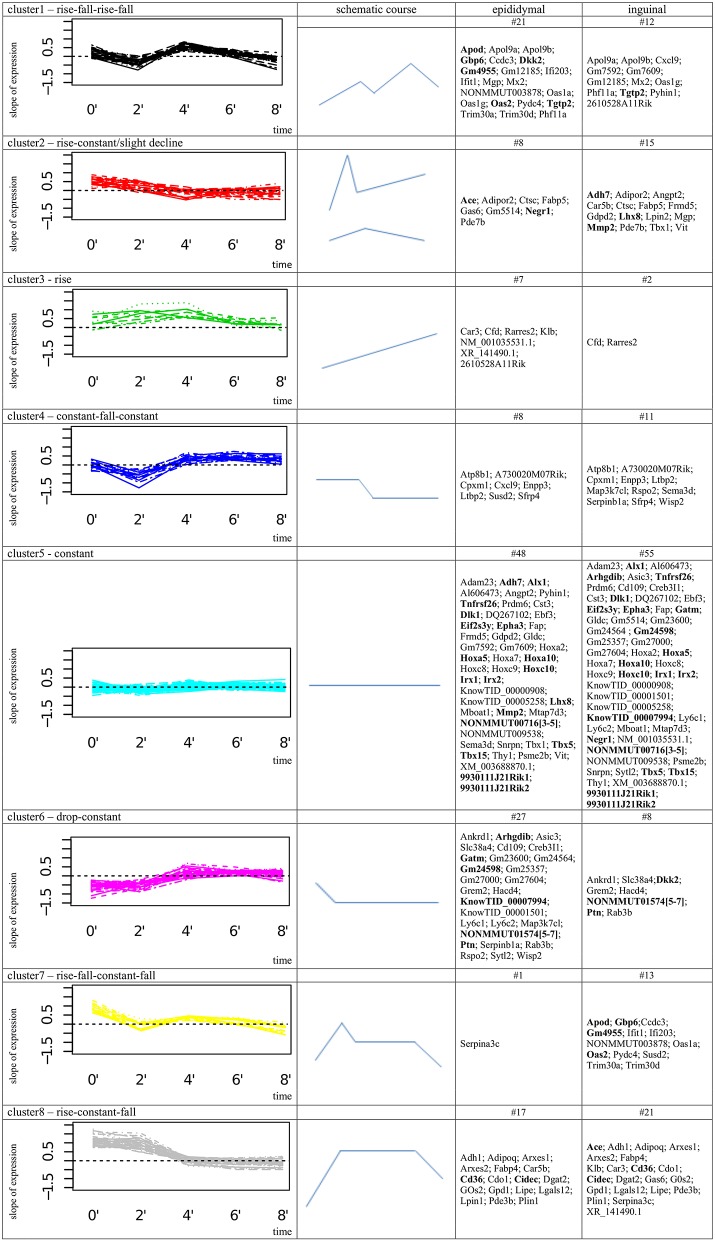
Cluster analysis of 137 most differentially expressed genes between epididymal and inguinal adipogenesis. In column 1 the figure shows the clusters of gene expression intensity slopes (x'_i_), the schematic shape of the according expression profile is provided in column 2; column 3; and 4 list the gene symbols assigned to the cluster in epididymal or inguinal cell line. # provides number of genes in single cluster corresponding to epididymal or inguinal cell line; Bold gene names highlight the top 34 genes.

Accordingly, each schematic expression pattern represents a general gene expression dynamic that can be described as: cluster1: rise-fall-rise-fall; cluster2: rise-constant/slight decline; cluster3: rise; cluster4: constant-fall-constant; cluster5: constant; cluster6: drop-constant; cluster7: rise-fall-constant-fall; cluster8: rise-constant-fall (Figure 2).

Subsequently, for each gene the combinations of clusters, the gene was assigned to during epi and ing adipogenesis, was extracted. Thus, in addition to detection of a mere difference in gene expression intensity it was possible to compare the gene expression dynamics between both depots. Of the 137 filtered genes, 77 genes fell into the same cluster and thus shared the same pattern of gene expression dynamics between epi and ing, i.e., the expression level, but not the general expression progression were different (Supplemental Table 6). Another 60 genes were assigned to different clusters in epi and ing, meaning that they follow a different gene expression dynamics during epi and ing adipocyte differentiation (Supplemental Table 7). Interestingly, of these 60 genes 31 genes were clustered in cluster5 (constant gene expression) for one cell line whereat they have been assigned to another cluster for the other cell line. This implied that the gene responds with altered gene expression to the differentiation stimulus in either epi or ing, while expression intensity remains unaffected in the other tissue (Figure 2, Supplemental Table 7).

Of the 34 top genes most (15 genes) were assigned to cluster5 in epi and ing (cluster5-cluster5 combination). This reflects a more or less constant level of expression of the gene over time in both fat depots (Figure 2). However, the gene expression intensity was considerably different between epi and ing. Another four genes were categorized into cluster 5-cluster 2 combinations. While cluster 5 reflects a mostly constant expression in one tissue, cluster 2 implies a rise in gene expression intensity following the differentiation signal in the other type of adipocytes. Furthermore, four genes had constant expression in ing cells (cluster5), while their expression clearly decreased following the differentiation signal in epi adipocytes (cluster6). Four more genes were categorized to the cluster1-cluster7 combination. Here, genes in both of these clusters showed clear changes in expression intensities over time. The expression of the epi genes in cluster1 could roughly be summarized as rise-fall-rise-fall and the profiles of the ing genes in cluster7 as rise-constant-slight-decrease. Further, the combination of cluster1 (rise-fall-rise-fall; epi) and cluster6 (drop-constant; ing) contained one gene (*Dkk2*), as well as cluster2 (rise-constant/slight decline; epi)-cluster8 (rise-constant; ing) combination (*Ace*). For the five remaining top genes the expression profiles of both fat depots were assigned to the same cluster, meaning that the pattern of gene expression change over time was similar, while the expression intensity differed: cluster1 (rise-fall-rise-fall) contains *Tgtp2*; cluster6 (drop-constant) lists *Ptn* and *lincRNA* [NONMMUT01574(5–7)]; cluster8 (rise-constant) includes *Cd36* and *Cidec* (Figure 2).

### Gene Set Enrichment Analysis—GO-Term and KEGG Pathways

**Figure 3:**
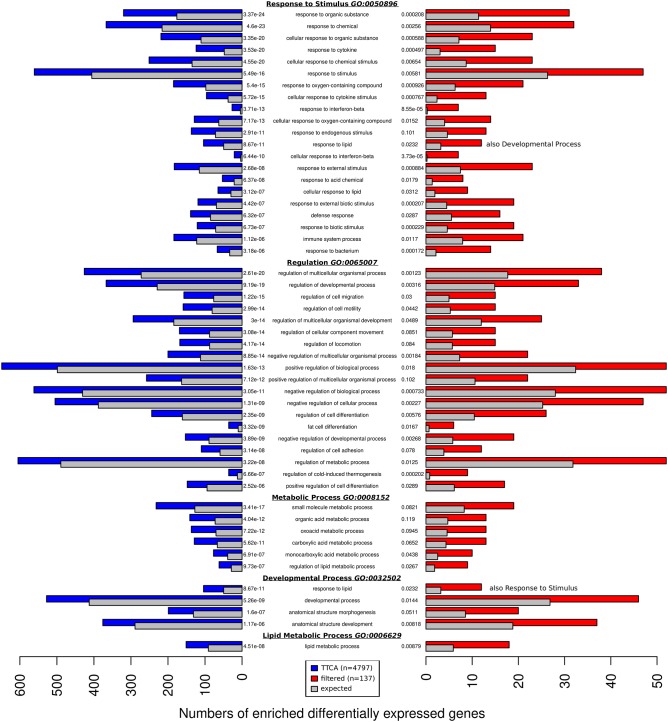
The overlap of the 50 most enriched GO terms found with genes reported by TTCA (4,797 blue) 137 filtered genes (red). The colored bars represent the number of genes assigned to the GO-term and compares it to the number of genes expected to be assigned to the term by chance (gray). The GO-terms can be categorized to five general biological processes: Response to Stimulus, Regulation, Metabolic Process, Developmental Process, and Lipid Metabolic Process. Each block is ordered by false discovery rate (FDR).

To identify significantly enriched processes, function, and pathways based on the differentially expressed genes during epi and ing adipogenesis we extracted significantly enriched GO terms for 137 filtered genes and 4797 TTCA genes. To reduce the over-interpretation of false positive enriched GO-terms we selected the 50 highest ranking hits in the overlap of both enrichments and summarized single processes according to their higher-level category. Thus, the following five main processes were identified: (i) Response to Stimulus (ii) Regulation Pattern Specific Process (regionalization, specification); (iii) Metabolic Process; (iv) Developmental Process, (i.e., morphogenesis, development); (v) Lipid Metabolic Process (Figure 3).

The KEGG pathway analysis uncovered eight pathways enriched with majority of the 137 differential expressed genes. In line with the GO terms, four of them were related to adipose tissue development [mmu04152: AMPK signaling pathway Supplemental Figures 4–5; mmu04923: Regulation of lipolysis in adipocytes (Supplemental Figures 6–7); mmu00561: Glycerolipid metabolism (Supplemental Figures 8–9); mmu03320: PPAR signaling pathway (Supplemental Figures 10–11)]. Three more were connected to cell infections [mmu05162: Measles (Supplemental Figures 12–13); mmu05160: Hepatitis C (Supplemental Figures 14–15); mmu05164: Influenza A (Supplemental Figures 16, 17)] and the remaining one to the nitrogen metabolism pathway [mmu00910: Nitrogen metabolism (Supplemental Figures 18–19)].

### Gene Expression and Interdepot Differences in Human Adipose Tissue

We analyzed the expression of the top 34 genes showing most divergent expression dynamics during adipogenesis in epi *vs*. ing cell lines in human vis and sc AT. Nine out of the 34 hits in epi/ing mouse cell lines do not present orthologs in primates or humans and/or were not present on the mRNA chips: *Gm4955*; *Gm24598*; *NONMMUT00716[3-5]*; *NONMMUT01574[5-7]*; *9930111J21Rik[1-2]*; *Tgtp2, KnowTID_00007994*; *Tnfrsf26*. Moreover, *LHX8* and *Gbp6* were not expressed in human AT at all.

**Figure 4:**
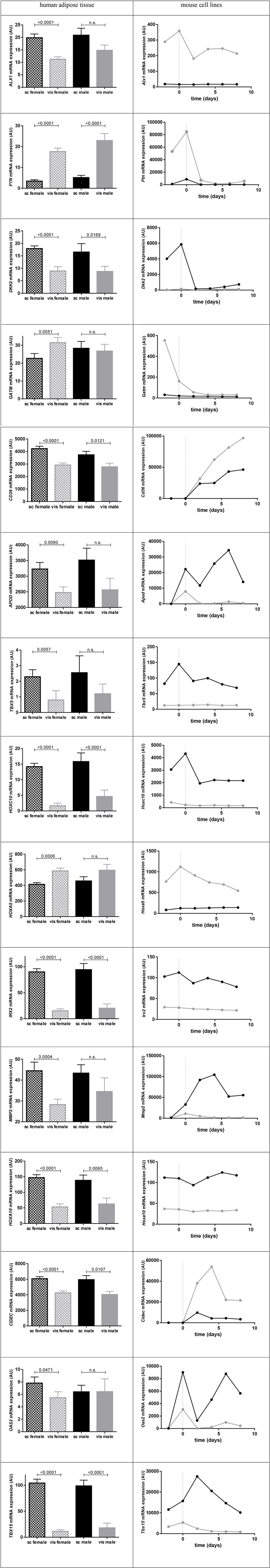
Gene expression of significant differentially expressed genes in human adipose tissue [*N* (females) = 47; *N* (males) = 16] and in mouse epididymal *vs*. inguinal cell line adipogenesis. On the left hand side data show subcutaneous (sc) and visceral (vis) adipose tissue gene expression means in males vs. females [arbitrary units (AU) ± standard error of the mean (SEM)]. *P*-values were calculated using Mann-Whitney-test. For *HOXA10* the NM_153715.1 transcript variant 1, *CD36* the NM_001001548 transcript variant 3 [transcript variant 1 presents almost same expression levels], for *ACE* the transcript variant 1 of NM_000789.2 and for *OAS2* the transcript variant 2 of NM_002535.2 is shown. On the right hand side data show mRNA expression in epididymal and inguinal mouse cell lines from 2 days before until 8 days after day of induction (dotted line; day 0). In black the mRNA expression of inguinal (corresponding to subcutaneous) and in gray the mRNA expression in epididymal (corresponding to visceral) cell line is shown.

Figure 4 presents the expression profiles of the remaining genes (*N* = 23), here the highest expression in humans was found for *CIDEC, CD36* (NM_001001548.1) transcript variants 1 and 3, *APOD, ARHGDIB, EIF2S3*, and *HOXA5*.

Comparing the gene expression levels in mouse cell line to the expression in human AT, three genes were contrarily expressed between the two species (*Alx1, Cd36*, and *Cidec*) (Figure 4, Supplemental Table 8).

### Gene Expression and Sex Differences in Human Adipose Tissue

Analyzing gene expression in mouse cell line *vs*. gene expression in gender stratified human AT we detected the same direction of expression in 21 out of 23 genes for both sexes whereat only *GATM* and *OAS2* presented gender specificity but this was marginal significant in females and not significant in males (Figure 4). In regard to the gene expression in mouse cell lines the non-stratified mean expression was same direction in mouse and humans (data not shown).

### Correlation of Human Gene Expression With Obesity and Related Traits

**Figure 5:**
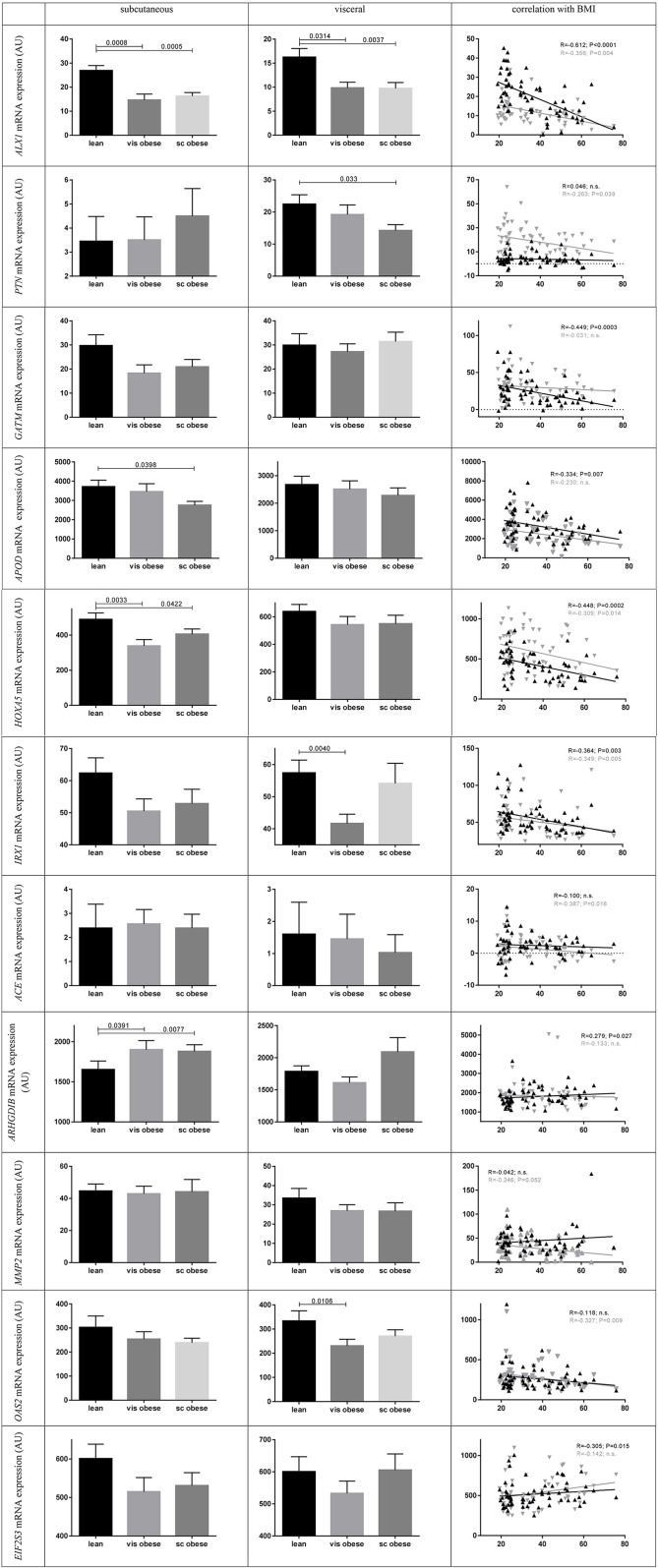
Human mRNA expression between BMI groups and correlation with BMI. Data present means ± standard error of the mean (SEM). For the lean BMI group 23 subjects were included, for visceral (vis) obese 17 and for subcutaneous (sc) obese 21. For correlation analysis black triangles and lines present subcutaneous and gray triangles and lines visceral mRNA gene expression (*N* = 63 paired samples). *P*-values were calculated using Mann-Whitney-test and Spearman's Rho correlations. For *ACE* the transcript variant 1 of NM_000789.2 and for *OAS2* the transcript variant 2 of NM_002535.2 is shown.

We found significant differences in mRNA expression between BMI groups for *ALX1, APOD, HOXA5*, and *ARHGDIB* in sc AT (Figure 5). Similarly, there were differences in vis AT according to BMI groups for *ALX1, OAS2* (NM_002535.2 transcript variant 2), *IRX1*, and *PTN* (Figure 5). Also correlational analysis supported these findings by showing correlations of BMI with sc mRNA expression of *ALX1, GATM, APOD, HOXA5, IRX1, EIF2S3*, and *ARHGDIB*, and with vis mRNA expression of *ALX1, PTN, HOXA5, IRX1, ACE, MMP2*, and *OAS2* (NM_002535.2 transcript variant 2) (Table 2). For WHR we found a correlation with *ACE* and *ARHGDIB* in sc AT only (all *P* < 0.05; Table 2).

**Table 2:** Correlation of BMI and WHR with the mRNA expression in human adipose tissue.

	**BMI**						**WHR**					
	**sc**			**vis**			**sc**			**vis**		
	***N***	***R***	***P***	***N***	***R***	***P***	***N***	***R***	***P***	***N***	***R***	***P***
*ALX1*	63	−0.612	**9.934** **×** **10**^**−8**^	63	−0.356	**0.004**	54	−0.233	0.089	54	−0.235	0.087
*PTN*	54	0.046	0.740	62	−0.263	**0.039**	47	0.154	0.211	53	0.151	0.281
*EPHA3*	62	−0.041	0.749	62	0.021	0.873	53	0.201	0.149	53	0.061	0.664
*DKK2*	63	0.085	0.508	61	0.065	0.616	54	0.051	0.716	53	−0.019	0.892
*GATM*	61	−0.449	**2.847** **×** **10**^**−4**^	63	−0.031	0.812	52	0.096	0.497	54	0.032	0.819
*NEGR1*	44	−0.142	0.357	45	−0.053	0.731	36	0.046	0.788	38	0.038	0.822
*CD36 TV1*	63	0.233	0.066	63	0.053	0.682	54	0.042	0.764	54	0.023	0.868
*CD36 TV3*	63	0.139	0.278	63	0.051	0.692	54	0.122	0.378	54	0.109	0.431
*APOD*	63	−0.334	**0.007**	63	−0.230	0.069	54	−0.001	0.993	54	0.150	0.280
*Tbx5*	50	0.021	0.884	37	0.035	0.836	43	−0.034	0.826	32	0.033	0.858
*HOXC10*	63	−0.151	0.237	38	−0.103	0.538	54	−0.065	0.640	33	0.078	0.667
*HOXA5*	63	−0.448	**2.314** **×** **10**^**−4**^	63	−0.309	**0.014**	54	−0.110	0.430	54	−0.055	0.693
*IRX1*	63	−0.364	**0.003**	63	−0.349	**0.005**	54	−0.091	0.512	54	−0.080	0.567
*ACE*	51	−0.100	0.484	38	−0.387	**0.016**	42	0.311	**0.045**	37	−0.043	0.799
*DLK1*	48	−0.164	0.265	45	−0.005	0.976	42	−0.177	0.262	36	0.108	0.531
*ADH7*	34	−0.297	0.088	27	0.252	0.204	31	−0.171	0.356	22	0.284	0.201
*IRX2*	63	−0.213	0.094	59	0.081	0.541	54	−0.083	0.550	51	0.126	0.378
*ARHGDIB*	63	0.279	**0.027**	63	−0.133	0.300	54	0.315	**0.020**	54	0.064	0.643
*MMP2*	63	−0.042	0.743	63	−0.246	**0.052**	54	−0.027	0.847	54	0.150	0.277
*HOXA10*	62	−0.161	0.211	62	−0.025	0.847	53	0.127	0.365	53	−0.125	0.372
*CIDEC*	63	−0.095	0.457	63	0.084	0.512	54	0.043	0.759	54	0.128	0.354
*OAS2*	63	−0.118	0.356	63	−0.327	**0.009**	54	−0.012	0.933	54	−0.123	0.374
*TBX15*	61	−0.246	0.056	52	0.068	0.633	52	−0.032	0.824	43	0.037	0.813
*EIF2S3*	63	−0.305	**0.015**	63	−0.142	0.267	54	−0.040	0.774	54	−0.127	0.361

*Spearman's Rho Correlations; For CD36 the NM_001001548 TV 1 & 3, for ACE the TV 1 of NM_000789.2, HOXA10 the NM_153715.1 TV 1, and for OAS2 the TV 2 of NM_002535.2 was used for analyses. BMI, body-mass-index; WHR, waist-to-hip ratio; sc, subcutaneous adipose tissue; vis, visceral adipose tissue; TV, transcript variant; bold values present P ≤ 0.05*.

## Discussion

In the present study, we tested the hypothesis that differences in adipogenesis in two fat depots of the human body are developmentally driven and related to the degree of obesity. We used a transcriptomic approach to gain mechanistic insight into the role of vis and sc fat in the metabolic disease by comparing the gene expression patterns of the vis and sc AT during differentiation of immortalized murine epi and ing adipocytes. According to GO-term and KEGG pathway analysis the majority of the 137 identified in time-course differentially expressed genes were enriched in pathways related to AT development. Finally, in human vis and sc AT, several genes exhibited variability in expression which correlated closely with BMI and/or WHR. To our knowledge, this is the first study that compares the transcriptome profiles of vis and sc AT during adipogenesis by using the recently reported TTCA algorithm to comprehensively study distinct depot-specific expression patterns.

Variation in body fat composition due to depot-specific accumulation of fat contributes to obesity-associated metabolic sequelae. In particular, larger amount of intra-abdominal fat manifested by higher WHR, is associated with higher risk for T2D and CVDs (46, 47). In contrast, lower WHR with larger amount of gluteal fat is linked to lower mortality, as well as lower risk for T2D and hypertension (48–50). There are several lines of evidence for a genetic background of body FD. It has been shown in twin and family based studies that body FD is a heritable trait ranging from 36 to 47% [reviewed in (11)], and also several GWAS clearly indicated that there are multiple genetic loci significantly associated with measures of body FD such as WHR or pericardial fat (51–53). In addition, the GWAS also pointed out that loci associated with ectopic fat might carry genes regulating adipocyte development and differentiation (12). In the present study TTCA revealed 137 genes differentially expressed between epi and ing AT over time. The top genes with most divergent expression dynamics during adipogenesis in epi *vs*. ing cell lines included developmental genes such as *Alx1, Lhx8, Hoxc10, Hoxa5, Irx1, Irx2, Hoxa10, Tbx15, and Tbx5*. Further, we found seven genes (*EPHA3, NEGR1, TBX5, HOXC10, IRX1, CIDEC, TBX15*) out of our top 34 hits that have also been shown to be associated with WHR and/or BMI in GWAS [according to the GWAS catalog (https://www.ebi.ac.uk/gwas/home)]. Thus, genetic differences between individuals may affect the expression or activity of these genes during adipogenesis and thereby maybe contribute to the risk of developing obesity or differential fat distribution. According to GO-term and KEGG pathway analysis, the majority of the 137 differentially expressed genes were enriched in four pathways related to AT development (AMPK signaling pathway; regulation of lipolysis in adipocytes; glycerolipid metabolism; PPAR signaling pathway).

The embryonic tissue development is based on three major lineages: (I) ectodermal, resulting in the formation of the brain and the peripheral nervous system; (II) endodermal, with the formation of organs like thymus, lung, pancreas, prostate, gastrointestinal tract, liver, and thyroid; and the (III) mesenchymal lineage which splits into paraxial mesoderm, resulting in skeletal muscle, dermis and bone formation, intermediate mesoderm, with formation of kidney, urinary tract and reproductive system, and the lateral plate mesoderm resulting in limb, spleen, smooth muscles, lymph, endothelium blood and adipose tissue. This is a highly complex system that needs to be well-regulated and balanced during differentiation processes. This complexity may at least partially explain the wide variety of the 137 pre-selected genes that were identified by TTCA and taken forward to pathway analyses. Our comparative approach between vis (epi) and sc (ing) AT suggested that the differentially expressed genes are mainly enriched in processes such as “developmental process,” “pattern specific process,” and “(lipid) metabolic process” which, according to the mouse genome informatics (MGI) database (http://www.informatics.jax.org/) (54), represent biological processes whose specific outcome is the progression of an integrated living unit like an anatomical structure or the creation of a defined area. Also further processes suggested by the analyses like “response to stimulus” and “regulation” appear to be in line with the assumed pathways, in particular when considering the experimental set up of adipogenesis *in vitro* characterized by stimulus-mediated differentiation of pre-adipocytes into adipocytes. Consistently, these findings are strongly reflected by KEGG pathway analyses pointing to genes belonging to the family of Hox-, T-box-, and iroquois (irx) genes (9%) that encode well-established transcription factors with known functions also in humans (55). The 137 significant genes contain 14 genes classified as homeobox genes, of which seven (*Alx1*; *Lhx8*; *Hoxa5*; *Hoxa10*; *Hoxc10, Irx1*; *Irx2*) rank among the 34 top genes. Serving as transcription factors regulating gene expression of many target genes, homeobox proteins are frequent key players during cellular differentiation including adipocytes, as supported by our findings as well.

In addition to the protein-coding developmental genes, several interesting non-coding candidates have been revealed in the present study. Small nucleolar RNA Gm24598 is assigned to the SNORD113/SNORD114 family, whose members are specifically expressed in brain and located in introns of the imprinted long non-coding RNA Rian (RNA Imprinted and accumulated in Nucleus) on mouse chromosome12. Gm24598 maps on chromosomeX overlapping the pseudogene retro-Rian (1,253 nts shorter than Rian). The human paralogous are encoded in two clusters of 9 and 31 copies in introns of the imprinted lncRNA MEG8 (maternally expresses gene 8) on chromosome 14. Members of the SNORD113/114 family comprise complementarity to regions on the 18S-rRNA, although these regions have not been proved to possess corresponding methylated residues, leaving their exact function still unknown. Other non-coding genes with a potential role in obesity identified in the present study are NONMUT00716(3–5), NONMUT01574(5–7), and KnownTID_00007994. They represent non-coding antisense and linc RNAs that are often assumed to have regulatory functions similar to transcription factors (56, 57).

Finally, it has to be acknowledged that, in line with our expectations, several established marker genes of adipogenesis like *CIDEC, CD36, APOD, ARHGDIB*, and *HOXA5* showed time-course related differences between vis and sc AT. These genes exhibited the highest expression in human AT out of the top 34 genes. *CIDEC* is a regulator of lipid metabolism including the regulation of lipid droplet size and stimulation of intracellular lipid deposition (58–60). Also *CD36* and *HOXA5* are known key players in adipocyte differentiation and adipogenesis (61, 62) supporting the present findings. Therefore, we are confident that the here generated data set reflects plausible and well-replicated genes and pathways related to adipogenesis, which underlines its strong potential in identifying depot-specific expression profiles.

Although for some of the top 34 hits in epi/ing mouse cell lines no orthologs have been identified in primates or humans, in addition to the highest expressed *CIDEC, CD36, APOD, ARHGDIB*, and *HOXA5*, developmental genes such as *ALX1, HOXC10, HOXA5, IRX1, IRX2, HOXA10*, and *TBX15* showed prominent expression in human AT. Moreover, similar to the data from epi/ing AT in mice, majority of these genes expressed differences between human vis and sc AT depots as well. These findings are in agreement with Gesta et al. (9) who reported developmental genes exhibiting dramatic differences in the level of expression in adipose and pre-adipose tissue from different regions of the body. However, similar to Gesta et al., the differential expression between fat depots was not the only feature of the identified genes (9), as several of the here identified genes including developmental genes (*HOXC10, HOXA5, IRX1*) showed strong correlation with BMI. Taking into account their known role in metabolic pathways, some of these genes may be of particular interest in obesity research. For instance, *APOD*, is an atypical apolipoprotein (63) supposed to be involved in the transport of lipophilic molecules and the active interference with their metabolism as well as signaling in an antioxidant and anti-inflammatory manner [reviewed in (64)]. *ARHGDIB*, another candidate for adipogenesis regulation is known to suppress metastasis (65) and to inhibit the endothelial axis and crosstalk with macrophages (66) (Supplemental Table 9).

We are aware that our study does not allow drawing conclusions regarding convincing events or pathways which are relevant for the development of function of the two distinct ATs. Nevertheless, we describe differential gene expression patterns and related networks to provide a basic platform for the identification and functional characterization of potential candidate genes and their encoded proteins involved in the development of distinct fat depots and associated with obesity.

In conclusion, our study revealed differences in time course related gene expression patterns in vis and sc AT during adipogenesis, thus providing novel insights into the molecular changes specific to the two main adipose types. In addition, in human vis and sc AT, several genes exhibited variability in expression which correlated closely with BMI and/or WHR, suggesting that fat depots-specific molecular mechanisms regulating adipogenesis may contribute to the individual risk of obesity.

## Data Availability Statement

The raw data supporting the conclusions of this article will be made available by the authors, without undue reservation, to any qualified researcher.

## Ethics Statement

The studies involving human participants were reviewed and approved by Ethic Commitee University Leipzig Geschäftstelle der Ethik-Kommission an der Medizinischen Fakultät der Universität Leipzig, Haus: Karl-Sudhoff-Institut für Geschichte der Medizin und der Naturwissenschaften, Käthe-Kollwitz-Straße 82, 04109 Leipzig. The patients/participants provided their written informed consent to participate in this study.

## Author Contributions

JB analyzed human data and wrote the manuscript. SK analyzed the chip data and did statistical analyses for epi/ing data. LM did the cell culture experiments. PS contributed to the statistical analyses of the manuscript. YB provided human expression data and contributed to the manuscript. MB is the PI for the human cohort used and contributed to the manuscript. MS and PK had the project idea and contributed to the manuscript.

### Conflict of Interest

The authors declare that the research was conducted in the absence of any commercial or financial relationships that could be construed as a potential conflict of interest.

## Supplementary Material

All Supplementary Material files are included in http://www.bioinf.uni-leipzig.de/supplements/18-054

## Abbreviations

AT: adipose tissue
BMI: body-mass index
cl: cluster
CVD: cardiovascular disease
epi: epididymal
FD: fat distribution
FFA: free fatty acids
GSEA: gene set enrichment analysis
GWAS: genome-wide association studies
ing: inguinal
KEGG: Kyoto Encyclopedia of Genes and Genomes
sc: subcutaneous
TG: triglycerides
TTCA: transcript time course analysis
T2D: type 2 diabetes
vis: visceral
WHR: waist-to-hip ratio.
